# 

**Published:** 1973-10

**Authors:** 


					Bristol Medico-Chirurgical Journal Vol. 88 (iiii) No. 328
Contents
The Development of Maternity Services in Bristol ...
C. C. Hancock
51
The Medical Library ... ... ... ... ... ... ... ... ... ... 57
News and Notes ... ... ... ... ... ... ... ... ... ... 58
Index ... ... ... ... ... ... ... ... ... ... ... 58
Printed by F. Bailey & Son Ltd., Dursley, Glos
& i. 7 S

				

